# Remembering Professor Walter A. Scott

**DOI:** 10.3390/v6103873

**Published:** 2014-10-20

**Authors:** Stefan G. Sarafianos

**Affiliations:** Departments of Molecular Microbiology & Immunology and Biochemistry, University of Missouri, 471d Bond Life Sciences Center 1201 E. Rollins Str., Columbia, MO 65211, USA; E-Mail: sarafianoss@missouri.edu; Tel.: +1-573-882-4338

**Keywords:** Walter Scott, HIV drug resistance

## Abstract

Walter Scott was a Biochemistry professor at the University of Miami, Miller School of Medicine and a leading figure in the field of HIV drug resistance. His untimely passing in January 2013 marked a loss for his family, as well as for students and colleagues who knew him as a dedicated and unassuming scholar, and a lively scientist with a great sense of humor.

Walter Scott was a Biochemistry professor at the University of Miami, Miller School of Medicine, and a leading figure in the field of HIV drug resistance. His untimely passing in January 2013 marked a loss for his family, as well as for students and colleagues who knew him as a dedicated and unassuming scholar, and a lively scientist with a great sense of humor.

I came to know Walter in the late 1990s, when he solved a long standing conundrum that had puzzled, for many years, biochemists, virologists, and clinical scientists: HIV patients treated with AZT were failing therapy through the selection of HIV reverse transcriptase mutations that could not be validated with biochemical assays. Walter discovered that this was because these mutations impart on HIV reverse transcriptase a novel activity that overcomes the chain-terminating activity of AZT, allowing for viral replication to resume. In seminal work, published in key papers, he showed that a common intracellular molecule, ATP, serves as an acceptor for this unblocking reaction. His surprising findings paved new ways of thinking about drug resistance and inspired countless experiments that led to biochemical and structural characterization of a novel major mechanism of viral drug resistance.

Walter Scott was born in Los Angeles, and raised in Oregon. After completing his Ph.D. in chemistry at the University of Wisconsin, he received post-doctoral training at the University of California at San Francisco and at Johns Hopkins University School of Medicine under the mentorship of Nobel Laureate Daniel Nathans. He joined the University of Miami in 1975 and started a molecular virology laboratory in the Department of Biochemistry and Molecular Biology. For many decades he led a well-funded laboratory, dedicated to important discoveries and the exceptional training of students who will remember him for his patience and wise mentorship.

In addition to his innovative ground breaking research, Walter Scott contributed to the scientific community by serving in many committees, including the AIDS Discovery and Development of Therapeutics NIH study section. All of us in this group fondly remember him as an exemplary conscientious reviewer of grants who was always fair, thorough, constructive, and supportive of young faculty.

Walter Scott will be missed by many friends in the HIV field, not only for his scientific wisdom, but also for his contagious laughter and gentle demeanor.

